# Postural responses to target jumps and background motion in a fast pointing task

**DOI:** 10.1007/s00221-018-5222-6

**Published:** 2018-03-23

**Authors:** Yajie Zhang, Eli Brenner, Jacques Duysens, Sabine Verschueren, Jeroen B. J. Smeets

**Affiliations:** 1 Department of Human Movement Sciences, Vrije Universiteit Amsterdam, Amsterdam Movement Sciences, Amsterdam, The Netherlands; 2 Department of Kinesiology, FaBer, KU Leuven, Leuven, Belgium; 3 Department of Rehabilitation Sciences, FaBer, KU Leuven, Leuven, Belgium

**Keywords:** Postural control, Visual perturbation, Two-step paradigm, Target jump, Background motion, Arm reaching

## Abstract

When reaching towards an object while standing, one’s hand responds very quickly to visual perturbations such as the target being displaced or the background moving. Such responses require postural adjustments. When the background moves, its motion might be attributed to self-motion in a stable world, and thereby induce compensatory postural adjustments that affect the hand. The changes in posture associated with a given hand movement response may, therefore, be different for the two types of perturbations. To see whether they are, we asked standing participants to move their hand in the sagittal direction away from their body to targets displayed on a horizontal screen in front of them. The target displacements and background motion were in the lateral direction. We found hand movement responses that were in line with earlier reports, with a latency that was slightly shorter for target displacements than for background motion, and that was independent of target displacement size or background motion speed. The trunk responded to both perturbations with a modest lateral sway. The two main findings were that the upper trunk responded even before the hand did so and that the head responded to background motion but hardly responded to target displacements. These findings suggest that postural adjustments associated with adjusting the hand movement precede the actual adjustments to the movement of the hand, while at the same time, participants try to keep their head stable on the basis of visual information.

## Introduction

When we reach out for objects in daily life, we often do not only adjust the angles of the joints of our arm. Sometimes, adjustments to the joints in the leg and trunk contribute directly to reaching the object, such as when stretching out and standing on one’s toes to reach something on a high shelf. If there is no direct contribution to bringing the hand to the object, we refer to adjustments as postural adjustments. Postural adjustments are frequently required for the control of balance (i.e., not falling over). This is, for instance, so when shifting one’s hips backward while leaning forward to reach a floating toy in a tub. In general, balance is challenged by moving the arm, while the efficiency and accuracy of the goal-directed arm movement are challenged by the postural requirements of maintaining balance (Berrigan et al. 2006). This relationship explains why arm movements are accompanied by anticipatory postural adjustments that are tuned to the requirements of the upcoming arm movement (Aruin and Latash 1995; Bouisset and Zattara 1987). Rapid postural adjustments are also observed when a moving arm is perturbed mechanically, so the link between arm movements and postural adjustments is not limited to planned aspects of the movement (Lowrey et al. 2017).

Another issue that we often have to deal with in daily life is adjusting an ongoing reaching movement. For instance, if we want to take a glass off a tray held by a waiter, we not only have to adjust our posture to keep balance while reaching for it, but might also have to adjust the whole movement as the tray moves. It is well known that arm movements can be adjusted when the target of the action is displaced (Georgopoulos et al. 1981; Pelisson et al. 1986; Soechting and Lacquaniti 1983). The trajectory can start to be adjusted 100–150 ms after the target is displaced (Brenner and Smeets 1997; Gritsenko et al. 2009; Kadota and Gomi 2010; Oostwoud Wijdenes et al. 2011; Oostwoud Wijdenes et al. 2013), and such adjustments occur even if the person in question is not aware of the change in target location (Goodale et al. 1986).

Our first question is whether such fast adjustments to the trajectory of the arm are accompanied by postural adjustments, and in particular whether such postural adjustments precede the adjustments to the arm, as they do in a simple reaction time task (e.g., Bouisset and Zattara 1981; Slijper et al. 2002). Given that adjustments have to be made as quickly as possible, having to first adjust one’s posture may result in the response having to be delayed, and, therefore, in longer latencies of the response (Slijper et al. 2002). Indeed, Leonard et al. (2011) found adjustments in electromyographic (EMG) activity of some postural muscles before adjustments in activity of the prime movers for a hand movement. In their study, the early postural adjustments were accompanied by a relatively long latency of the hand movement adjustment (> 175 ms). Alternatively, rather than delaying the response of the hand, the postural adjustments might only occur after the onset of the correction, as has been reported for choice reaction time tasks (Slijper et al. 2002) and for responses to mechanical perturbations of the arm (Lowrey et al. 2017).

A final issue that we might have to deal with is that there may be movement in the background, such as many people walking by as we try to take the glass off the tray. There are numerous reports of goal-directed arm movements being influenced by motion in the surrounding (Brenner and Smeets 1997; Saijo et al. 2005; Whitney et al. 2003). A possible reason for quickly responding to motion in the background is that such motion is attributed to self-motion that requires a postural response (Gomi 2008; Mergner et al. 2005). That motion in the surrounding would influence postural responses is consistent with peripheral visual information playing an important role in postural stability when standing (Nashner and Berthoz 1978). However, the response of the arm to background motion is not mainly guided by peripheral visual information (Brenner and Smeets 2015). The latency of responses of the arm to background motion is 110–160 ms (Brenner and Smeets 1997; Gomi et al. 2006; Whitney et al. 2003), which is similar to that for target jumps (100 to 150 ms; Brenner and Smeets 1997; Gritsenko et al. 2009; Kadota and Gomi 2010; Oostwoud Wijdenes et al. 2011; Oostwoud Wijdenes et al. 2013). The magnitude of the response depends on the magnitude of the target displacement and on the speed of background motion (Brenner and Smeets 1997; Saijo et al. 2005).

Our second question is whether postural responses to the target being displaced are similar to the postural responses to background motion. If the postural response is in anticipation of the arm movement adjustment, we expect to see very similar postural responses for similar arm movement responses. However, if the background motion induces a postural response that includes an arm movement, possibly to compensate for the suggested self-motion, we might at least initially expect the response to be quite different from the postural response that is made to maintain one’s balance when adjusting the arm movement after a target is displaced. Although we expect a clearly different response in the latter case, it might be that the trunk also responds before the hand in a purely postural response.

To answer our questions, we first verify that fast adjustments of reaching movements when the target of the reach is displaced are accompanied by anticipatory postural adjustments (Leonard et al. 2011). We call any response before the onset of the hand adjustment ‘anticipatory’. We then examine whether the postural response to background motion is in anticipation of the arm or is an independent response by comparing the postural adjustments in response to target jumps and background motion in conditions with similar adjustments to the arm movement. Answering the two questions will contribute to a better understanding of the interaction between manual and postural control when one is standing and reaching.

## Methods

### Participants

Sixteen right-handed participants (28.3 ± 3.0 years, 7 males) participated in the experiment. They had normal or corrected-to-normal vision. None of the participants had any disease that can affect motor or sensory function. The study was approved by the Research Ethics Committee of the Faculty of Behavioural and Movement Sciences, Vrije Universiteit Amsterdam. Written informed consent was obtained from each participant.

### Experimental setup

**Fig. 1:**
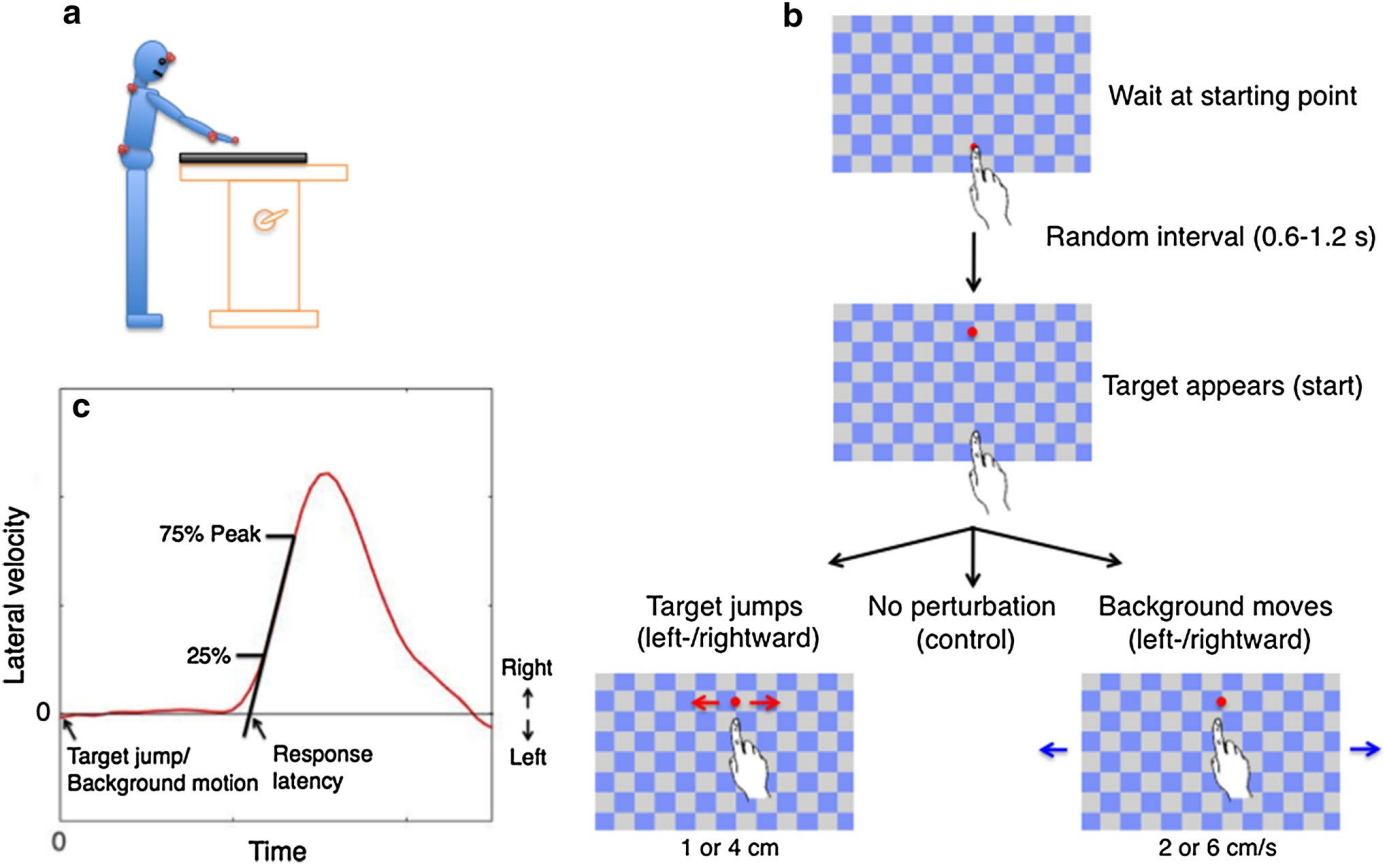
Methods. **a** Schematic side view of a participant in the experimental setup, with the red discs indicating the marker positions. **b** Sequence of visual events in the three types of trials. **c** Velocity profile of a typical lateral response with latency determination using the extrapolation method

A screen (60 Hz refresh rate, 91.9 × 51.6 cm, 1920 × 1080 pixel resolution) was positioned horizontally on a table (Fig. 1a). The participant stood barefoot with his or her feet separated by about 10% of the participant’s height, 15 cm from the near edge of the screen. The height of the table could be adjusted to align the screen with the participant’s hip. A photodiode was attached to the upper right corner of the screen to detect when the target appeared and when it changed position or the background started moving (with an error of 5 ms at most).

An Optotrak 3020 motion capture system (Northern Digital, Waterloo, Ontario, Canada) with two cameras was used. One camera was behind the participant and one to his or her right. The sampling rate was 200 Hz. The posture was recorded with customised cluster markers: three markers attached rigidly to each other in a triangular configuration. Cluster markers were attached to the forehead, 3rd thoracic vertebra (referred to as ‘upper trunk’), 1st sacral vertebra (referred to as ‘lower trunk’) and the wrist (ulnar side). A single marker was attached to the nail of the index finger of the right hand. The latter was used to control the experiment on the basis of the movement of the finger.

### Experimental task and procedure

Each session started with a calibration procedure for determining the position of the index finger when in contact with given positions on the screen in Optotrak coordinates. After the calibration, participants started individual trials by moving their right index finger to the starting point. The starting point of each arm movement was a dot (radius: 1 cm) that was shown on the checkerboard-like background (square length: 7 cm). Once the index finger had been resting on the starting point for a random time between 0.6 and 1.2 s, the starting point disappeared and the target dot (radius: 1.5 cm) appeared. The participant was instructed to tap on the target as accurately and quickly as possible with the tip of the right index finger.

While the participant moved towards the target, either target displacement or background motion could occur, triggered by the finger having moved 5 mm from the starting point on the screen. Due to delays in measuring the movement of the finger and rendering images on the screen, the actual perturbation was 60 ms after the finger crossed this threshold. If the target was hit (i.e., if the contact position of the finger, as determined by the calibration, was within the target), a sound indicated success. Otherwise, the target drifted away from where the finger touched the screen.

The experiment consisted of nine conditions: four target jump conditions, four background motion conditions, and a no-perturbation condition. In the target jump conditions, the target jumped either 1 or 4 cm, leftwards or rightwards, across the stationary background. In the background motion conditions, the background moved either leftwards or rightwards at 2 or 6 cm/s, ‘below’ the stationary target. We chose perturbation sizes that would be likely to make the magnitude of the response of the hand comparable for target jump and background motion conditions. This was based on a pilot study, in which we varied the target jump amplitude and background velocity. There were 300 trials in total: 30 trials each in four target jump conditions and four background motion conditions, and 60 trials in the no-perturbation condition. All trials were presented in a completely random order. The participants practiced for about 20 trials (random conditions) before the start of the experiment. During the experiment, they could rest at any time they liked by not moving to the starting point.

### Data analysis

The 3D kinematic data of all markers were filtered using a second order low-pass Butterworth filter with a cut-off frequency of 30 Hz. We determined this cut-off frequency by determining the minimum variance in the distances between the three markers on a cluster (Schreven et al. 2015). We found this minimum for frequencies between 20 and 30 Hz, depending on the body part. As the variance increases considerably for cut-off frequencies below the optimum frequency, and only mildly for higher frequencies, we chose 30 Hz to filter all lateral motion data (finger, wrist, head, upper trunk and lower trunk). We checked whether the choice for this cut-off frequency influenced our results by re-analysing the data with a 50 Hz cut-off frequency. The effects on observed latencies were less than 2 ms.

Movement time was calculated as the duration between the onset of the finger movement (finger lifted higher than 5 mm) and the finger touching the screen again. We excluded trials (5%) for which the duration or the delay in presenting the perturbation was not within ± 3SD of the mean, or for which the moment of the perturbation could not be determined properly (on the basis of the signal picked up by the photodiode). Movements to the right and away from the body were considered positive.

As the perturbations were always in the lateral direction, we only analysed responses in the lateral direction. The lateral velocity of the finger was calculated from the measured position data using the central difference algorithm. We defined time zero as the moment at which the perturbations actually happened, which was about 60 ms after the finger had been raised 5 mm from the screen. Responses were determined by comparing movements after rightward and leftward perturbations (differences between the movements in the direction of the perturbation were considered positive). Since this gives the sum of the responses in both directions, we divided the difference between the responses to rightward and leftward perturbations by two. The result is equivalent to a response to a rightward perturbation.

We used the extrapolation method to determine the latency of responses to the perturbations: the latency was the time at which a line through the points at which the response reached 25% and 75% of the peak response intersected a baseline value (Fig. 1c; Veerman et al. 2008). The baseline value was the average velocity from 50 ms before to 50 ms after the perturbation. This is possible because basing the response on the difference between trials with rightward and leftward perturbations removes any systematic lateral motion (or angular velocity) that is not related to the perturbation. The extrapolation method requires a clearly identifiable peak. As the data of some participants showed multiple peaks, using the individual responses would have forced us to exclude the data of some participants. The responses of body parts other than the finger were very modest with respect to the spontaneous trial-to-trial variability, so it was impossible to reliably identify response peaks for all individual participants. Therefore, we determined the latencies based on the average response of all participants. We bootstrapped (resampled) the trials within each participant to obtain a measure of reliability. We averaged the resampled responses of all participants and determined the latency for the average response. Doing so 1000 times provided a distribution of latencies based on resampled trials, which we used to determine a Bayesian 95% credible interval.

The cluster markers not only allow us to determine the lateral motion, but also rotations. Although we do not have predictions for the rotations, the azimuthal rotation might be informative. Despite optimal filtering at 30 Hz, the filtered signal turned out to be quite noisy. Fast Fourier transformed data revealed a peak in the spectrum of rotations that was absent in the translations (possibly due to cluster vibration). To present interpretable data on rotational movement, we had to filter the orientations at 10 Hz. Since this additional filtering smooths the responses considerably, we did not analyse the orientation data quantitatively.

Descriptive data are shown as means or means ± standard deviations (SD) across participants. The movement times were calculated for each trial, and then averaged across the four target (or background) conditions for each participant.

## Results

**Fig. 2:**
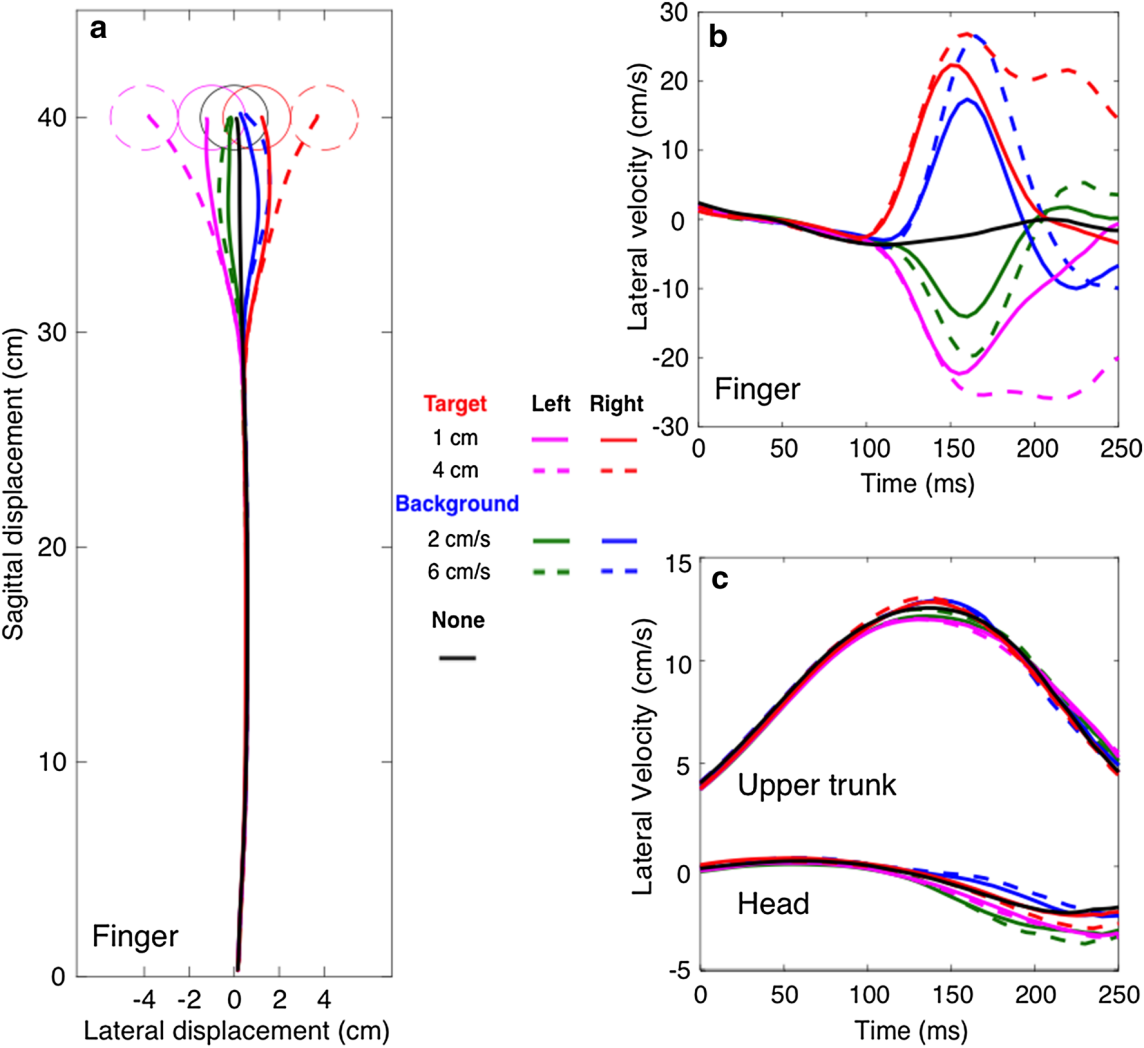
**a** Overview of the average finger paths of all participants in the nine conditions. The origin is the location of the starting dot. **b, c** Lateral velocity of the finger, upper trunk and head as a function of the time since the perturbation (about 60 ms after movement onset)

The mean movement times were 382 ± 44 ms for unperturbed trials, 400 ± 44 ms for trials with target jumps and 396 ± 43 ms for trials with background motion. Participants hit the target in 95.0 ± 3.9% of the 300 trials. This means that participants were able to successfully follow the target jumps and compensate for their initial responses to background motion (Fig. 2a). The finger always initially moved in the direction of the perturbation, regardless of whether the target jumped or background moved (Fig. 2b). If the background moved (blue and green traces), the finger followed the background motion even though the target was stationary, and it moved further in the direction of the perturbation for faster background motion (dashed blue and green traces). If the target jumped (red and magenta traces), the finger followed the target jump. The response reached a higher peak velocity and lasted longer for a larger target displacement (dashed red and magenta traces). The responses of both the upper trunk and the head are small in comparison to the lateral movements that occur even without any perturbation (Fig. 2c). We checked for a possible postural response to the perturbation in the anterior–posterior direction. No postural adjustments were found within 200 ms after the perturbation.

**Fig. 3:**
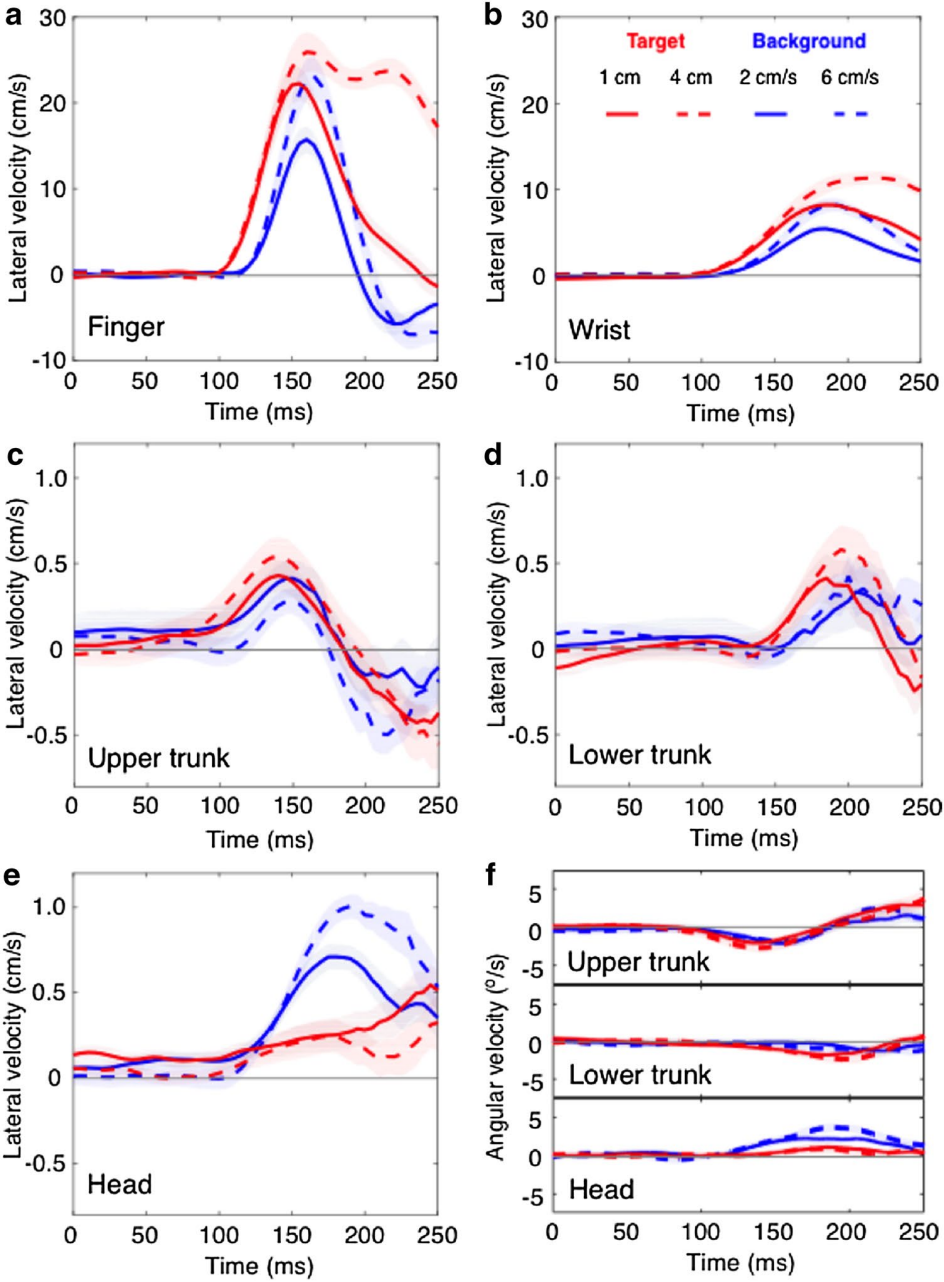
Average response to target jumps and background motion as a function of the time since the perturbation for the **a** finger, **b** wrist, **c** upper trunk, **d** lower trunk and **e** head. **f** Response in azimuthal angular velocity for the upper trunk, lower trunk and head (clockwise is positive). Shaded areas represent the SEM across participants. Note that the scales for responses of the hand (**a, b**) are different from those for the body (**c, d, e**)

Concentrating on the lateral responses, we find that the finger and wrist showed similar magnitudes of responses when the target jumped as when the background moved (compare red and blue traces in Fig. 3a, b). The trunk responses were much smaller than those of the wrist and finger, but also similar for the two types of perturbation (Fig. 3c, d). Remarkably, the head responded very different to target jumps than to background motion (Fig. 3e): there was hardly any initial response of the head to target jumps, whereas there was a clear response to background motion. The rotation of the head within 250 ms after the perturbation was small, but the angular velocities were bigger in response to background motion than in response to target jumps (Fig. 3f). The azimuthal rotation of the upper and lower trunk also started earlier for target jumps than for background motion (Fig. 3f).

**Fig. 4:**
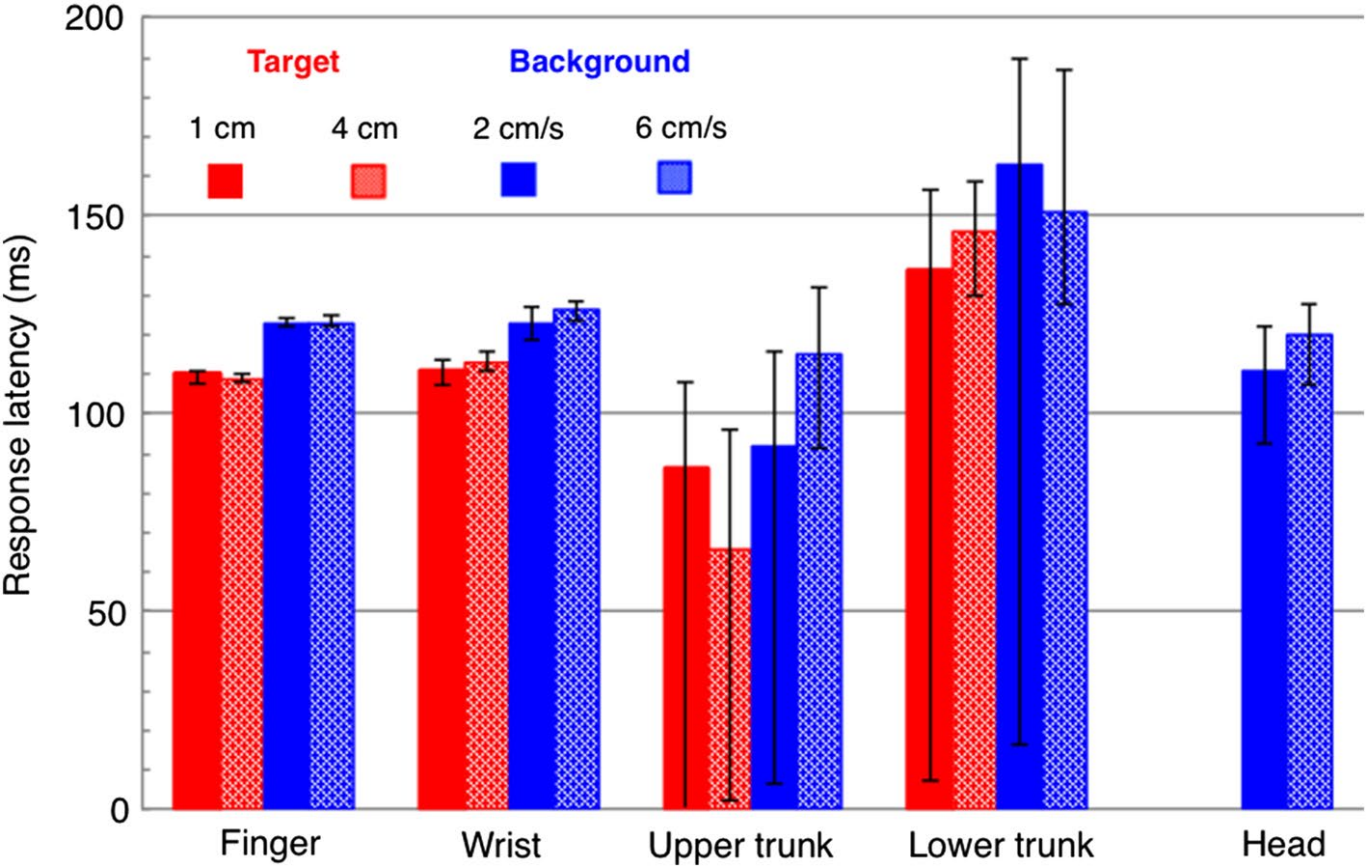
Response latencies of different body parts to target jumps and to background motion. The bars represent latencies calculated from the mean curves shown in Fig. 3. The error bars show Bayesian 95% credible intervals. As the bootstrapped data were noisy, the extrapolation method sometimes yielded nonsensical (negative) values for the latencies. We included these nonsensical values in the determination of the credible interval

Perturbation size (target jump; background velocity) did not affect the hand response latencies. The latencies of the lateral responses to the target jumps were shorter than those to the background motion for the finger (109 vs. 123 ms), as well as the wrist (112 vs. 124 ms; Fig. 4). The latency difference between target jumps and background motion conditions varied between 14 and 28 ms for different body parts (Fig. 4), which is (given the filtering at 30 Hz) within the temporal precision of determining the latency according to the extrapolation method based on velocity (Oostwoud Wijdenes et al. 2014). The head responded to the background motion after about 116 ms. Notably, the upper trunk was the first part of the body to respond to the perturbations. It responded about 76 ms after the target jumps, about 33 ms before the hand responded (Fig. 4). The lower trunk responded about 32 ms later than the hand.

## Conclusions and discussion

We compared the responses of manual and postural adjustments to two types of perturbations (target jump and background motion). Both the perturbations and the responses were in the medio-lateral direction. The upper trunk moved in the same direction as the perturbation, before the hand did so. The postural adjustments to the two types of perturbation were similar for all parts of the body that we measured, except the head. One limitation of the study is that we only use trunk and head displacements as a measure of anticipatory postural adjustments, although some muscle activations may control posture without leading to observable displacements. This limitation would have made the interpretation of a null-finding problematic, but does not hamper the interpretation of the clear anticipatory postural adjustments that we observed.

We interpret the very early upper trunk response as an anticipatory postural adjustment. The reason is that it occurred about 33 ms before the response became apparent in the hand, and thus did not contribute directly to moving the hand. Both when the target jumped and when the background moved, the first response was in the upper trunk (at the shoulder level; Fig. 4). The response was in the same direction as the subsequent movement of the arm, suggesting that it was made in anticipation of the rotation of the shoulder and elbow. Trunk movements in a direction that matches a future arm movement have been found before when there is the possibility of a perturbation (Martin et al. 2000). The fastest responses have been found in the leg (Fautrelle et al. 2010; Leonard et al. 2011) and at the shoulder level when adjusting to a target jump in depth (Fautrelle et al. 2010). In that case, it was the EMG activity in the muscle of the shoulder rather than the displacement of the shoulder that was earlier. That it took tens of ms longer for the wrist to respond than for the upper trunk to do so, makes it unlikely that this just has to do with a shoulder-to-wrist sequence. Thus, the answer to our first question is *yes*, arm responses to target perturbations are preceded by anticipatory postural adjustments.

We did not observe differences in the responses of the hand and trunk to the two types of perturbation. In contrast, the head clearly responded to background motion, but hardly responded to target jumps. The difference in head response between the two types of perturbations that resulted in similar responses of hand and trunk suggests that there is a separate cause for the responses of the head. An obvious way to interpret this is that participants were trying to keep their head stable relative to the surrounding when adjusting the reach (which makes sense for arm movements in a stable environment). Following this reasoning, the head motion induced by moving the background is likely to be a reaction to the motion of the background being attributed to self-motion (see introduction). Hence, there are different postural responses to target jumps and background motion, but there are also common components to the postural responses, which answers our second question.

We found no effects of target jump size on hand response latencies, which is in line with earlier research (Brenner and Smeets 1997; Veyrat-Masson et al. 2010). We also found no effect of background velocity on hand response latency, in line with earlier research (Saijo et al. 2005). The latencies of finger responses to target jumps were 14 ms shorter than those to background motion in the present study. In the previous studies with both target jumps and background motion, the responses to target jumps had a 40-ms shorter latency (Brenner and Smeets 1997) or a 15 ms *longer* latency (Kadota and Gomi 2010) than those to background motion. These differences may be caused by differences between the setups or stimuli, because the attribute that defines the target can influence the response latency (Veerman et al. 2008), as might the contrast, pattern and size of the background. Brenner and Smeets (1997) used faint lines as their background, while Kadota and Gomi (2010) and we used high-contrast checkerboards. The high contrast might result in quicker response latencies.

The difference in latency suggests that the responses to the two types of perturbations rely on different pathways. The background-induced responses are likely to involve cortical pathways. Global motion, such as the background motion in our experiment, activates many visual areas (Palmisano et al. 2015), including some that appear to be specialised in analysing optic flow (such as VIP, the ventral intraparietal area; Schaafsma and Duysens 1996; Gabel et al. 2002a, b). It has been suggested that the fast corrections of hand movements might rely on subcortical mechanisms (Day and Brown 2001). This could explain the latency difference. However, the posterior parietal cortex is also considered to be involved in fast online adjustments to target displacements, because a patient with bilateral posterior parietal lesions could not make such corrections (Pisella et al. 2000), and transcranial magnetic stimulation (TMS) of the contralateral parietal cortex disrupts fast corrections (Desmurget et al. 1999). Moreover, fast online adjustments can be mediated by the parvocellular pathway (Brenner and Smeets 2003; Veerman et al. 2008) and can occur even if responding requires the processing of attributes such as colour (Brenner and Smeets 2004; Veerman et al. 2008). Therefore, whether the use of different pathways is responsible for the latency difference is not clear.

In conclusion, when visual perturbations occur during a hand movement while standing, there are postural adjustments that precede the manual adjustments. These postural adjustments are very similar, but not identical, when the target of the action is displaced and when the background moves. The main difference is to be found in the movement of the head.
